# Immunogenomic intertumor heterogeneity across primary and metastatic sites in a patient with lung adenocarcinoma

**DOI:** 10.1186/s13046-022-02361-x

**Published:** 2022-05-11

**Authors:** Runzhe Chen, Jun Li, Junya Fujimoto, Lingzhi Hong, Xin Hu, Kelly Quek, Ming Tang, Akash Mitra, Carmen Behrens, Chi-Wan Chow, Peixin Jiang, Latasha D. Little, Curtis Gumbs, Xingzhi Song, Jianhua Zhang, Dongfeng Tan, John V. Heymach, Ignacio Wistuba, P. Andrew Futreal, Don L. Gibbons, Lauren A. Byers, Jianjun Zhang, Alexandre Reuben

**Affiliations:** 1 Department of Thoracic/Head and Neck Medical Oncology, the University of Texas MD Anderson Cancer Center, Houston, TX 77030 USA; 2 Department of Genomic Medicine, the University of Texas MD Anderson Cancer Center, Houston, TX 77030 USA; 3 Department of Radiation Oncology, Sun Yat-sen University Cancer Center, State Key Laboratory of Oncology in South China, Collaborative Innovation Center for Cancer Medicine, Sun Yat-sen University, Guangzhou, 510060 Guangdong China; 4 Regor Therapeutics, 2445 Main Street, Second Floor, MA 02142 Cambridge, USA; 5 Department of Translational Molecular Pathology, the University of Texas MD Anderson Cancer Center, Houston, TX 77030 USA; 6 Department of Pathology, the University of Texas MD Anderson Cancer Center, Houston, TX 77030 USA

**Keywords:** Lung adenocarcinoma, Intertumor heterogeneity, Genomic, T cell repertoire

## Abstract

**Background:**

Lung cancer is the leading cause of cancer death, partially owing to its extensive heterogeneity. The analysis of intertumor heterogeneity has been limited by an inability to concurrently obtain tissue from synchronous metastases unaltered by multiple prior lines of therapy.

**Methods:**

In order to study the relationship between genomic, epigenomic and T cell repertoire heterogeneity in a rare autopsy case from a 32-year-old female never-smoker with left lung primary late-stage lung adenocarcinoma (LUAD), we did whole-exome sequencing (WES), DNA methylation and T cell receptor (TCR) sequencing to characterize the immunogenomic landscape of one primary and 19 synchronous metastatic tumors.

**Results:**

We observed heterogeneous mutation, methylation, and T cell patterns across distinct metastases. Only TP53 mutation was detected in all tumors suggesting an early event while other cancer gene mutations were later events which may have followed subclonal diversification. A set of prevalent T cell clonotypes were completely excluded from left-side thoracic tumors indicating distinct T cell repertoire profiles between left-side and non left-side thoracic tumors. Though a limited number of predicted neoantigens were shared, these were associated with homology of the T cell repertoire across metastases. Lastly, ratio of methylated neoantigen coding mutations was negatively associated with T-cell density, richness and clonality, suggesting neoantigen methylation may partially drive immunosuppression.

**Conclusions:**

Our study demonstrates heterogeneous genomic and T cell profiles across synchronous metastases and how restriction of unique T cell clonotypes within an individual may differentially shape the genomic and epigenomic landscapes of synchronous lung metastases.

**Supplementary Information:**

The online version contains supplementary material available at 10.1186/s13046-022-02361-x.

## Background

Lung cancer is the leading cause of cancer death, partially owing to its extensive heterogeneity [1, 2]. It has been proposed that this extensive heterogeneity results from successive clonal expansion and selection of the fittest clones influenced by genomic accumulation and somatic epigenetic alterations [3–6]. However, tumor evolution may also be shaped by pressure from the immune system, which can prune the most immunogenic branches of the tumor [7].

T cells play a crucial role in preventing cancer development through antigen-specific detection and destruction of malignant cells, though evolving tumors can eventually escape immune surveillance through a process termed immunoediting [4, 8–11]. Few studies have addressed the impact of the T cell repertoire in shaping metastatic heterogeneity [12–14] with most work to date evaluating longitudinal changes spanning multiple timepoints and therapies. Though these studies offer crucial insights, they do not allow the evaluation of intrinsic intertumor heterogeneity in absence of selective pressure from therapy. Furthermore, to date, the analysis of intertumor heterogeneity has been limited by an inability to concurrently obtain tissue from synchronous metastases unaltered by multiple prior lines of therapy [15–17].

Here, we sought to study the relationship between genomic, epigenomic and T cell repertoire heterogeneity in a rare autopsy case from a 32-year-old female never-smoker with left lung primary late-stage lung adenocarcinoma (LUAD) with more than 20 synchronous metastases. We observed heterogeneous mutation, methylation, and T cell patterns across distinct metastases including a set of prevalent T cell clonotypes which were completely excluded from left-side thoracic tumors. Our work further highlights neoantigen methylation as a potential mechanism driving immunosuppression and some of the hurdles facing the treatment of late-stage lung cancer.

## Methods

### Human subject research

We collected 20 tumor samples and one normal gastrointestinal (GI) tract sample at autopsy. Collection and use of patient samples were approved by the Institutional Review Board of the University of Texas MD Anderson Cancer Center. Clinical information is presented in Supplementary Table 1.

### Sample collection

DNA of collected samples was isolated from FFPE tissues using the AllPrep DNA/RNA FFPE Kit (Qiagen, Hilden, Germany). Hematoxylin and eosin (H&E) slides of each case were reviewed by experienced lung cancer pathologists under the microscope to assess the percentage of tumor tissues versus normal tissues. Tumor cell viability was also assessed by examining the presence of necrosis in the tissues. Only samples with enough viable tumor cells were selected for whole-exome sequencing (WES), methylation and immunoSEQ.

### DNA preparation

Unstained tissue sections (10 μm thick) were deparaffinized in xylene and 100% ethanol (twice in each for 10 minutes). The macrodissected tumor areas of the deparaffinized tissues were placed into 1.5 mL collection tubes for DNA extraction. The tissue was next suspended with Buffer PKD and Proteinase K from the Allprep FFPE kit. After incubating at 56 °C for 15 min and on ice for 5 min, the mixed solution was centrifuged for 15 minutes at 20,000 x g. Finally, the DNA samples were quantified by Nano Drop 1000 Spectrophotometer (Thermo Scientific, Wilmington, DE, USA). The fragmentation sizes were evaluated by the Agilent 2200 Tape Station system using the Genomic DNA Screen Tape Assay (Agilent Technologies, Santa Clara, CA, USA).

### Whole-exome sequencing

Exome capture was performed on 500 ng of genomic DNA per sample based on KAPA library prep (Kapa Biosystems) using the Agilent SureSelect Human All Exon V4 kit according to the manufacturer’s instructions and paired-end multiplex sequencing was performed on the Illumina HiSeq 2000 sequencing platform. The average sequencing depth was 178x (ranging from 63x to 225x, standard deviation +/− 31).

### Mutation calling

Tumor contents and major/minor copy number changes were estimated by Sequenza (v2.1.2) [18]. To control those FFPE caused artifact contaminations, somatic single nucleotide variants (SNVs) was first called using MuTect version 1.1.4 [19], VarScan 2 [20] and Strelka2 [21] with default setting, respectively. Then, the following filtering criteria were applied to each callers: 1) sequencing depth ≥ 20× in tumor DNA and ≥ 10 × in germline DNA; 2) variant allele frequency (VAF) ≥ 0.02 in tumor DNA and < 0.01 in germline DNA; 3) the total number of reads supporting the variant calls is ≥4; and 4) variant frequency is <0.01 in ESP6500, 1000 genome and EXAC databases. Mutations called by Mutect with a LOD score < 10 were further filtered out, and those mutations called by Strelka with a quality score below 35 was also filtered out. Finally, only those mutations were kept if called by any of the two tools and rescued if any were rejected but shared by at least two tumors. Identified missense mutations were manually reviewed using the Integrative Genomics Viewer (IGV) version 2.3.61 [22, 23].

### Phylogenetic analysis

Ancestors were germline DNA assuming with no mutations. The phylogenetic tree was generated as described [24]. A binary presence/absence matrix of all somatic mutations detected was used as input for R package phangorn version 2.0.2 [25].

### Neoantigen predictions

Nonsynonymous mutations were identified from WES profiling and the binding affinities with patient-restricted MHC Class I molecules of all possible 9- and 10-mer peptides spanning the nonsynonymous mutations were evaluated with the NetMHC3.4 algorithm based on HLA-A, HLA-B, and HLA-C alleles of each patient [26–28]. Candidate peptides were considered as HLA binders when IC50 < 500 nM with high affinity binders presenting IC50 < 50 nM.

### DNA methylation profiling and tumor–immune microenvironment deconvolution

Genomic DNA (approximately 500 ng) was bisulfite converted using EZ DNA Methylation Kit (Zymo Research Corp. Irvine, CA, USA) following the manufacturer’s protocol. Bisulfite converted DNA materials were then processed and hybridized to the Infinium Human Methylation 450 k arrays (Illumina, San Diego, CA, USA) according to manufacturer’s recommendation. Preprocessing and initial quality assessments of the raw data were examined using the following R packages. Subset-quantile within-array normalization (SWAN) [29] was used to normalize raw methylation values. IlluminaHumanMethylation450k.db annotation package was used to annotate the CpG probes location. Probe sets with a beta value above 0.3 were taken as methylated [30, 31]. Before any genomic and statistical analyses were conducted, we normalized and inspected the methylation data for the presence of substantial confounding batch effects. Cellular deconvolution analyses were carried out using estimated cellular fractions using MethylCIBERSORT [32].

### TCRβ sequencing and comparison parameters

Immunosequencing of the CDR3 regions of human T cell receptor (TCR) β chains was performed using the protocol of immunoSEQ (Adaptive Biotechnologies, hsTCRβ Kit) [33–35]. Two sets of PCRs were performed on DNA extracted from the tissues collected. The initial PCR used a mix of multiplexed V- and J-gene primers which amplify all possible recombined receptor sequences from the DNA sample, and a second PCR designing to add unique DNA barcodes to each PCR product was followed. After that, samples were pooled together with a negative and a positive control. The pools were then sequenced on an Illumina MiSeq platform using 150 cycle paired-end protocol and sequence-ready primers. After finish the sequencing, the raw data were transferred to Adaptive Biotechnologies and processed into a report including those passed quality-check samples and a normalized and annotated TCRβ profile repertoire accordingly. Profile of TCR rearrangements is presented in Supplementary Data.

T-cell density in FFPE tissue samples was calculated by normalizing TCR-β template counts to the total amount of DNA usable for TCR sequencing, where the amount of usable DNA was determined by PCR-amplification and sequencing of housekeeping genes expected to be present in all nucleated cells. T-cell richness is a metric of T cell diversity, and it is calculated by on the T-cell unique rearrangements. T-cell clonality is a metric of T cell proliferation and reactivity, and it is defined as 1-Peilou’s evenness and is calculated on productive rearrangements by:

$$1+\frac{\sum \limits_i^N{p}_i{\log}_2\left({p}_i\right)}{\log_2(N)}$$

where p_i_ is the proportional abundance of rearrangement i, and N is the total number of rearrangements. Clonality ranges from 0 to 1: values approaching 0 indicate a very even distribution of frequency of different clones (polyclonal), whereas values approaching 1 indicate a distinct asymmetric distribution in which a few activated clones are present at high frequencies (monoclonal). JI is conceptually a percentage of how many objects of two sets have in common out of how many objects they have in total. JI = (number of rearrangements in common) / (total number of rearrangements). Statistical analysis was performed in R version 3.2. 

### Statistical Analysis

Graphs were generated with GraphPad Prism 8.0. Spearman’s rank correlations were calculated to assess the association between 2 continuous variables. Kruskal-Wallis tests were used for categorical variables with more than 2 levels. *P*-values less than 0.05 were considered to be statistically significant.

## Results

### Patient information

**Fig. 1:**
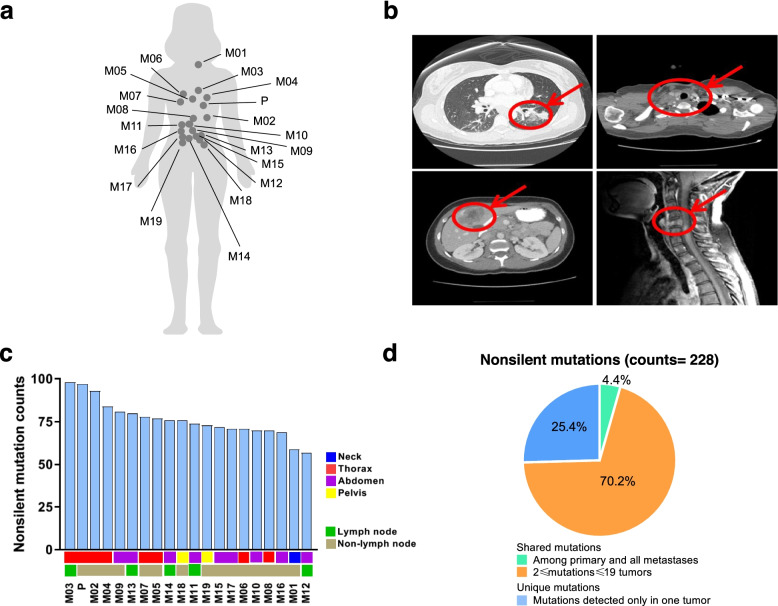
Synchronous metastatic tumors exhibit heterogeneous growth and somatic mutation and neoantigen patterns. **a** Anatomical map of representative biospecimen collection sites in the patient. **b** Representative CT and MRI scans of different resected tumors: primary lung tumor, cervical lymph node enlargement, liver metastasis and C5 spine compression fracture by metastasis. **c** Non-silent mutation counts in tumors. **d** Fraction of shared and unique non-silent mutations across tumors

A 32-year-old female never-smoker presented to her primary care physician complaining of weakness in her upper right arm lasting for two weeks. Physical examination was unremarkable, other than grade 3 weakness in her right upper limb. Shortly after, she was hospitalized due to acute venous thromboembolism of this arm. She was started on anti-coagulants and underwent computed tomography (CT) scans of the chest, abdomen and pelvis and magnetic resonance imaging (MRI) of the brain as part of the work up. Numerous nodules were detected suggestive of extensive metastasis and a 1.9 cm left lung mass was consistent with a lung primary origin (Fig. 1a, b). A liver biopsy revealed the diagnosis of poorly differentiated LUAD. Core biopsy of left chest wall nodule also revealed poorly differentiated LUAD with tumor cells positive for pan-keratin and TTF-1 and negative for ER, PR, CDX2, WT1, PAX-8, synaptophysin and chromogranin. The patient underwent palliative radiation therapy for C5-C7 spine metastases with 10 × 300 cGy and one dose of chemotherapy with carboplatin and paclitaxel while awaiting molecular profiling results. Her condition deteriorated rapidly and she expired 13 days following her sole dose of chemotherapy due to the disseminated metastatic cancer and cancer-associated complications including severe respiratory, cardiovascular and renal failure caused by Trousseau syndrome and embolic stroke despite continuing hydrocortisone combined with antibiotic therapy for the last two days preceding her death. An autopsy was performed and widely disseminated metastatic carcinoma involving multiple systems and organs was observed.

To understand the genomic and T cell landscape of this extensively metastatic LUAD, 20 tumor samples (Supplementary Table 1) including the left lung (primary tumor, P), thyroid gland (M01), left pleural cavity (M02), left hilar lymph node (M03), left parietal pleura (M04), heart (M05), right lung (M06), right pleural cavity (M07), 12th thoracic vertebra (M08), gastrointestinal (GI) tract (M09), liver (M10), 4 abdominal lymph nodes (M11–14), left adrenal gland (M15), two metastases in the right kidney (M16 and M17), left and right ovaries (M18 and M19) as well as one histologically normal sample from the GI tract were collected and subjected to WES, DNA methylation array and TCR sequencing.

### Distinct mutational profiles are seen across primary tumor and synchronous metastases

Overall, 228 non-silent mutations were detected with an average of 76 per sample (range = 57–98). The number of non-silent mutations varied between tumors, with only 10 shared across all 20 samples (Fig. 1c). Of these non-silent mutations, 170 (75%) were shared by at least two tumors while 58 (25%) were unique (Fig. 1d). When canonical cancer gene mutations were analyzed [36–38], commonly-mutated cancer genes included TP53, CDKN2A, ASXL1 and MET in this patient (Supplementary Fig. 1a). Only TP53 mutation (chromosome 17_7578382, stop gain, spectrum G- > C) was detected in all tumors suggesting TP53 mutation was an early genomic event, while other cancer gene mutations were later events which may have followed subclonal diversification. We also constructed a phylogenetic tree to depict the genomic heterogeneity and evolutionary trajectory of these metastatic tumors. As shown in Supplementary Fig. 1b, the phylogenetic structure varied considerably between tumors highlighting profound genomic heterogeneity within this patient. We then utilized the Jaccard index (JI), which takes into consideration the proportion of shared non-silent mutations between any two samples. The JI ranged from 0.14 to 0.82 (average = 0.49) with more proximal tumors generally more genetically similar (Supplementary Fig. 1c). Homology between the primary and metastases ranged from 0.14 to 0.73 (average = 0.33), with the thoracic lesions including the left pleural metastasis, left hilar lymph node and right pleural metastasis exhibiting the greatest similarity with the primary tumor. Taken together, these results reveal marked genomic heterogeneity across different metastases within the same patient.

### The T cell infiltrate in distant metastases is more dense, diverse and reactive

**Fig. 2:**
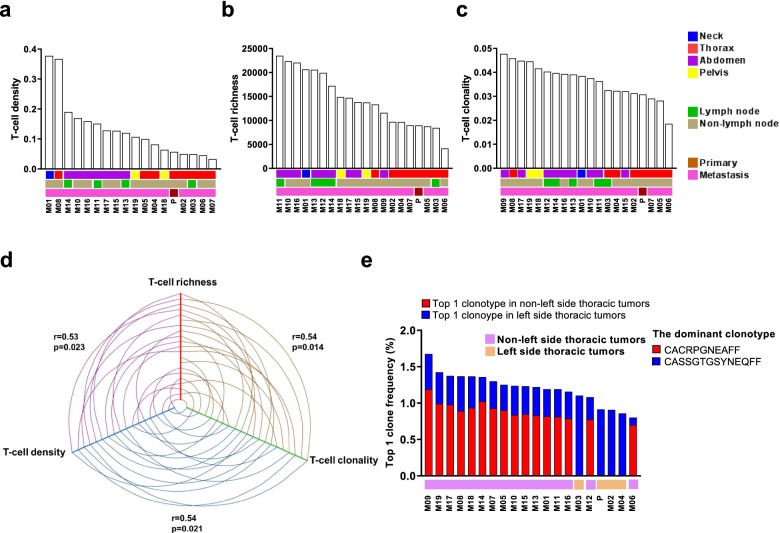
Characterization of T cell repertoire metrics across tumors. T-cell **a)** density, **b)** richness and **c)** clonality. **d** Correlation between T-cell density, richness and clonality. **e** Distribution of most prevalent TCR clonotype. The dominant TCR clonotype in left-side thoracic tumors (P, M02, M03 and M04) was CASSGTGSYNEQFF, while the dominant TCR clonotype in non-left thoracic tumors was CACRPGNEAFF

The crucial role of T cells in immunoediting led us to study the T cell repertoire to further investigate the spatial heterogeneity of T-cell responses [39, 40]. T-cell density, an estimate of the proportion of T cells within a tumor, ranged from 3 to 38% (average = 13%, Fig. 2a), while richness, a measure of T-cell diversity, ranged from 4,168 to 23,487 unique T-cell rearrangements (average = 14,344 unique rearrangements, Fig. 2b). T-cell clonality, a measure of T-cell reactivity, ranged from 0.02 to 0.05 (average = 0.04, Fig. 2c). All TCR metrics were positively inter-correlated (Density *vs.* Richness: r = 0.53, *p* = 0.02; Density *vs.* Clonality: *r* = 0.54, *p* = 0.02; Clonality *vs.* Richness: *r* = 0.54, *p* = 0.01, Fig. 2d). Compared to prior work from our group in an early-stage LUAD cohort [41], lower T-cell density (*p* < 0.05) and clonality (*p* < 0.0001) and higher richness (*p* < 0.0001) were observed in tumors from this patient (Supplementary Fig. 2a-e).

Tumors were then grouped anatomically. Non-thoracic tumors displayed higher T-cell density (*p* < 0.01), richness (*p* < 0.0001) and clonality (*p* < 0.01) than thoracic tumors (Supplementary Fig. 3a-c), perhaps owing to their anatomical location away from the primary tumor and its immunosuppressive influences [42, 43]. Lymph nodes serve as sites of T cell priming, activation and modulation, leading us to speculate that the interaction between metastatic cancer cells and T cells in lymph nodes may be distinct compared to other sites of metastases. However, no statistical differences were observed in relation to lymph node involvement (Supplementary Fig. 3d-f). Taken together, these data suggest differences in T cell response based on anatomical site, that is, T cell exclusion, suppression and a more focused T cell response in proximity to the primary tumor.

### Distinct T cell repertoire profiles are associated with left-side thoracic tumors

To evaluate T-cell responses in the tumors, we next focused on the most prevalent TCR clonotypes. Distinct clonotypes were detected in left-side thoracic tumors (left lung tumor, left pleural cavity, left hilar lymph node and left parietal pleura) compared to others. This result was intriguing considering bilateral pneumonia and leukocytosis were observed, though more inflammation was noted in the left thoracic cavity than the right. Strikingly, the most prevalent clonotype in “other” tumors (*CACRPGNEAFF*) was entirely undetectable in left-side thoracic tumors (P, M02, M03 and M04) (Fig. 2e). Similar trends were also observed among the top 5 and 10 TCR clonotypes with certain clonotypes completely excluded from left-side thoracic tumors (Supplementary Fig. 4a-b). These data illustrate spatial restriction even among the most prevalent T cell clonotypes across synchronous metastases.

### T cell repertoire heterogeneity is observed across differentially growing tumors

**Fig. 3:**
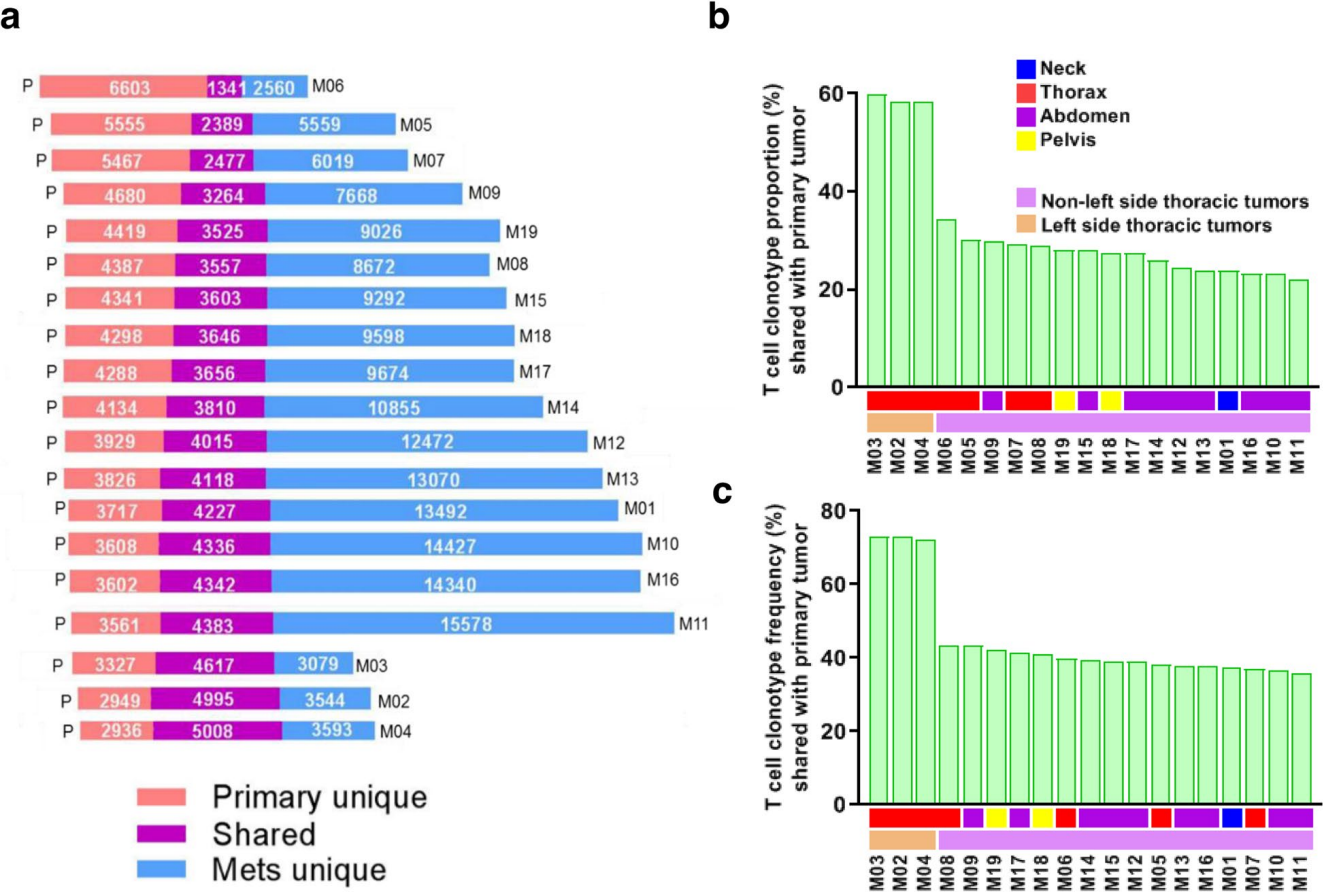
T cell repertoire heterogeneity is observed across differentially growing tumors. **a** Number of T cell clonotypes in the primary tumor (red), metastases (blue) or shared (purple). **b** Shared T cell clonotype proportions and **c)** frequencies between the primary tumor and metastases

**Fig. 4:**
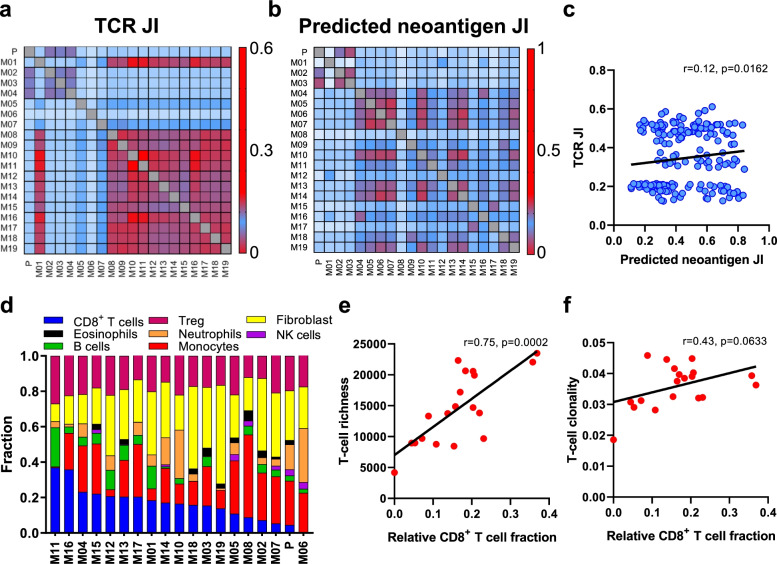
Evolution of synchronous metastases may be shaped by the T cell repertoire. **a** Quantification of T cell repertoire heterogeneity across tumors by Jaccard index (JI). **b** Quantification of predicted neoantigen heterogeneity across tumors by JI. **c** Correlation between T cell repertoire JI and predicted neoantigen JI. **d** Deconvolution of immune components and T cell subpopulations by MethylCIBERSORT. **e** Correlation between T-cell richness and estimated CD8^+^ Tcell fraction. **f** Correlation between T-cell clonality and estimated CD8^+^ Tcell fraction

To gain deeper insights into TCR heterogeneity, we assessed the overlap between T cell repertoires across different tumors. We first compared the proportion (JI) and frequency of T cell clonotypes shared between the primary tumor and metastases. In accordance with the unique T cell clonotype pattern observed in tumors from the left side thorax, proportions and frequencies of shared T cells were much more similar between the three left thoracic metastases (M02, M03 and M04) and primary tumor (P) (Fig. 3a-c). T cell repertoire heterogeneity was evident across all tumors, with an average JI value of 0.35 (ranging from 0.12 to 0.61) and more shared T cells between proximal tumors (Fig. 4a), significantly higher than in a previously published cohort of 11 multi-region localized non-small cell lung cancers (NSCLCs) [44] (average 0.35 *vs.* 0.17, *p* < 0.0001) (Supplementary Fig. 5). Next, we studied the proportion and frequency of shared T cell clonotypes across all 20 tumors. In total, 599 T-cell clones were shared across all tumors, with proportions ranging from 3.0 to 15.4% (average = 5.39%) and frequencies accounting for 11.9% to 21.5% of the T cell repertoire (average = 15.96%) (Supplementary Fig. 6a-b). Of interest, both a greater proportion (*p* < 0.01) and percentage (*p* < 0.01) of shared T cell clones were observed in thoracic tumors compared to non-thoracic tumors (Supplementary Fig. 6c-d).

### Evolution of synchronous metastases may be shaped by the T cell repertoire

**Fig. 5:**
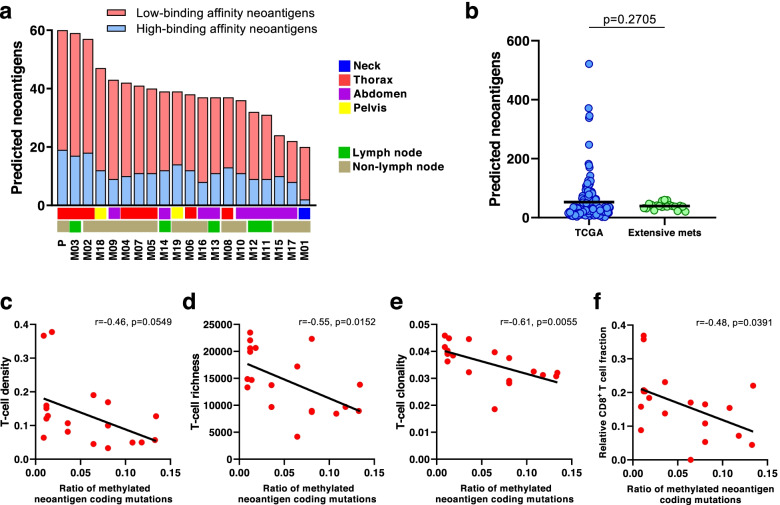
Methylated neoantigen burden is inversely correlated with T cell repertoire metrics. **a** Total number of predicted neoantigens across tumors. **b** Comparison of predicted neoantigens between TCGA cohort and tumors in our study. Correlation between ratio of methylated neoantigen coding mutations and T-cell **c)** density, **d)** richness, **e)** clonality and **f)** relative CD8^+^ T cell fraction

We next performed *in silico* prediction of HLA-A-, −B-, and -C-presented neoantigens using NetMHC3.4 [26–28]. On average, 39 predicted neoantigens (IC_50_ < 500 nmol/L) were detected per tumor, with the most (n = 60) seen in the primary tumor and fewest (n = 20) in the thyroid gland. Only 11 high binding affinity neoantigens were detected on average (IC_50_ < 50 nmol/L) with the most (n = 19) also in the primary tumor and least (n = 2) in the thyroid gland (Fig. 5a). This falls within range but below the average of 53 predicted neoantigens seen in non-smokers from TCGA (Fig. 5b). We then evaluated the relationship between the T cell repertoire and predicted neoantigens. Predicted neoantigen heterogeneity was also evident, with the average JI value of 0.44 (ranging from 0.11 to 0.84, Fig. 4b), and a weak but statistically-significant positive correlation between T cell repertoire and neoantigen homology (*r* = 0.12, *p* = 0.0162, Fig. 4c), which could suggest the distribution of T cells may be partially driven by their reactivity to shared neoantigens. Interestingly, ratio of methylated neoantigen coding mutations was negatively associated with T-cell density (*r* = −0.46, *p* = 0.0549), richness (*r* = −0.55, *p* = 0.0152) and clonality (*r* = −0.61, *p* = 0.0055) (Fig. 5c-e), suggesting neoantigen methylation may contribute to immune suppression and potentially explaining the weak neoantigen associations with T cell repertoire homology.

To assess immune infiltration, we next performed cellular deconvolution analyses using MethylCIBERSORT (Fig. 4d) [32]. Unfortunately, no distinct immune cell infiltration patterns were noted in left-side thoracic tumors (P, M02, M03 and M04) (Supplementary Fig. 7). However, relative CD8^+^ T cell fraction was correlated with richness (*r* = 0.75, *p* = 0.0002) and clonality (*r* = 0.43, *p* = 0.063) (Fig. 4e, f). Furthermore, CD8 to Treg ratio, which correlates with a more favorable outcome in cancer [45, 46], was also correlated with T-cell richness (*r* = 0.68, *p* = 0.0012) and clonality (*r* = 0.64, *p* = 0.032) (Supplementary Fig. 8a-b). A negative correlation between CD8^+^ T cell fraction and methylated neoantigen coding mutations was also observed (*r* = −0.48, *p* = 0.0391, Fig. 5e). These results highlight the greater proliferative potential of CD8^+^ T cells and suggest T cell reactivity and diversity may be mainly driven by the clonal expansion of CD8^+^ T cells at the patient level, as previously suggested by our group and others [47]. Overall, our findings suggest that the evolution of synchronous metastases may be shaped by the T cell response in absence of prior therapy.

## Discussion

Metastasis is an evolutionary process shaped by the dynamic interactions between tumor cells and host factors including immune surveillance [48]. T cells play a pivotal role in mediating this process by recognizing antigens presented on MHC molecules at the surface of tumors and carrying out cytotoxic responses against tumor cells harboring their cognate antigens [49]. Accordingly, much importance has been attributed to T cell infiltration in many solid tumors, with more T cells generally associated with a better prognosis [39, 50, 51]. However, recent studies have highlighted the impact of intratumor heterogeneity (ITH) and bystander T cells [52–54], and suggested that only ~10% of tumor-infiltrating lymphocytes are capable of recognizing antigens presented by the tumor they have infiltrated [55], prompting deeper investigations into the T cell repertoire. Our understanding of the role of genomic and immune heterogeneity in lung cancer has evolved in recent years, thanks to investigations by our group and others into differences between regions of individual tumors, synchronous metastases and between primary and metastatic tumors [44, 56–59] highlighting potential spatial and temporal factors influencing clinical outcomes [60, 61]. Here, we assess the characteristics of the T cell repertoire in a treatment-naïve non-smoking patient with synchronous lung metastases and depict the interplay between the primary tumor and synchronous metastases [11, 44, 62], revealing extensive immunogenomic intertumor heterogeneity across primary and metastatic sites.

In our study, clonal *TP53* mutations were detectable in all tumors, suggestive of an early genomic event, in line with prior reports [24, 63]. Interestingly, a higher overlap in somatic mutations was observed across proximal tumors suggesting they are more genetically similar, potentially due to metastatic seeding from the primary tumor [64, 65]. Though our study focused on a single patient, the overlap in mutational burden observed between synchronous metastases is in line with previous reports in lung [59], melanoma [66], kidney [67] and colon cancer [68]. Considering the role of somatic mutations in triggering T cell responses through the generation of neoantigens, this overlap suggests these somatic mutations may serve as potential therapeutic targets for vaccination or T cell engineering through targeting of unifying antigens present across all synchronous tumors. This is supported by the modest but significant correlation between shared mutations and shared TCRs though additional studies are needed to confirm these hypotheses.

We observed lower T cell repertoire heterogeneity across synchronous metastases in our study than in our prior work assessing multi-region ITH of localized LUAD [44]. This difference could highlight the distinct resistance mechanisms at play in accelerated progression in our study versus more gradual progression in early-stage LUAD which may have allowed for divergent genomic evolution and immune editing over years. This is reinforced by the absolute restriction of certain T cell clonotypes to metastases surrounding the primary tumor, which could be reflective of the distinct antigenic environments established in distal tumors. Unfortunately, our lack of deep immune phenotyping data precludes our ability to further investigate the role the distinct immune microenvironments, including chemokine gradients and receptors, which may have played a role in establishing these vastly distinct T cell microenvironments. However, the presence of shared T cell clonotypes could also be indicative of common responses against unifying antigens displayed across synchronous metastases.

Aberrant methylation has been reported to be involved in tumorigenesis in a variety of cancers and its impact on anti-tumor immune surveillance are very complex [69–72]. DNA methylation could affect immune surveillance directly by regulating the expression of potential neoantigens and/or immune-related genes thereby suppressing anti-tumor immune responses [73] or indirectly via modifying chromosomal vulnerability for mutations and copy number alterations (CNAs), both of which are well known to influence the tumor immune microenvironment [74–76]. A high level of hypomethylation could also lead to a high CNA burden which has been associated with a cold tumor immune microenvironment therefore facilitating immune evasion. Conversely, a high level of hypomethylation is associated with an increased mutational burden, which could increase tumor immunogenicity [74]. However, in the end, the selected cancer cell clones of late-metastases could escape immunosurveillance through the cumulative effects of these molecular aberrations. Recently, considerable progress has been reported in the identification of protein factors with a highly conserved DNA interaction surface, termed the methyl CpG-binding domain (MBD) [77]. MBD proteins could couple DNA methylation to transcriptional repression therefore silencing tumor suppressor genes that are hypermethylated at their promoter CpG islands in cancer cells [77]. MBD1 silences the IRF8 gene, which is a central mediator in the IFNγ/STAT1 signaling pathway governing the establishment of immune responses [78]. Therefore, disrupting MBD1-mediated silencing could enhance immune surveillance. Interestingly, in the current study, the ratio of methylated neoantigen coding mutations was negatively associated with T-cell density, richness and clonality, even at an individual level. One could therefore hypothesize that therapeutic agents modulating methylation could potentially reprogram the immune microenvironment and could exhibit some potential in treating these tumors.

Our study does exhibit certain limitations, including its focus on a single patient. However, analysis of several synchronous tumor sites from a single patient with advanced disease in absence of heavy pre-treatment is rarely possible due to the lack of clinical indication. Unfortunately, deeper analysis of underlying mechanisms, immune cells and soluble factors influencing T cell trafficking and heterogeneity remain unclear due to the archival nature of these samples and will require further investigation. It is well known that DNA methylation is involved in differentiation and activation of naïve CD4^+^ T cells [79, 80]. DNA methylation could release gene suppression upon demethylation of promoter regions regulating differential Th1 and Th2 cytokine secretion and therefore interrupting CD4^+^ T-cell polarization with effector phenotypes in these cells [81, 82]. Interestingly, up to 27% of methylated genes in promoter regions are actively expressed in naïve CD4^+^ T cells and associated with immune response and T cell differentiation [83]. Moreover, a recent study by Bam M *et al* [84] found that the majority of differentially methylated regions (DMRs) in tumor CD4^+^ T cells are located in the promoters followed by intergenic regions and introns. One could therefore assume that methylated genes in intergenic regions are also involved in naïve CD4^+^ T cell polarization and differentiation into specific lineages. Unfortunately, we were unable to investigate the impact of DNA methylation on the immune system and how genes in promoters or intergenic regions regulate CD4^+^ T cell differentiation and activation in our cohort due to a lack of metastasis-paired normal tissue controls.

Despite these limitations, our study provides important evidence of differential tumor-immune responses co-existing in metastases within the same individual, related not only to molecular alterations. As a result, our findings may also partially explain the challenge of treating late-stage lung cancer due to the heterogeneity of metastases. Additional genomic, transcriptomic and immune studies in patients with synchronous metastases could help shed light on these and other mechanisms at play and provide therapeutic insights into late-stage NSCLC. Lastly, our study demonstrates heterogeneous immunogenomic profiles across synchronous metastases and provides important evidence of differential tumor-immune responses co-existing in metastases within the same individual, partially resulting in the challenge of treating late-stage lung cancer. These results advocate for combination regimens with drugs exhibiting distinct mechanisms of action, e.g. concurrent chemotherapy with immunotherapy or anti-PD-1 plus anti-CTLA-4 [85] to eradicate cancer cells displaying different immunogenomic features to improve survival of patients with synchronous lung cancer metastases [66]. Ideally, biopsies of multiple sites should be considered when feasible to identify common neoantigens across different disease sites, which could be targeted therapeutically. Interestingly, a shared TP53 mutation was identified across all tumor lesions and the patient was found to express HLA-A*02:01. Recent work has revealed that TP53-derived neoantigens can be targeted using Bispecific T cell Engagers (BiTEs), highlighting a potentially effective therapeutic option for this patient [86]. Under most circumstances, however, multi-site biopsy is not clinically safe or practical. Alternatively, canonical cancer gene alterations, which are often present in all cancer across difference sites [87], are appropriate targets. Moreover, with the caveat of variable detection sensitivity, liquid biopsy is less impacted by tumor heterogeneity [88, 89], and may therefore provide a holistic view of molecular features across different metastatic sites to complement molecular profiling from tumor biopsies.

## Conclusion

In summary, in this study, we present the heterogenous immunogenomic landscape of one primary tumor and 19 synchronous metastases from a minimally-pretreated young female never-smoker with late-stage LUAD. Only a TP53 mutation was detected in all tumors suggesting it was an early genomic event while other cancer gene mutations occurred at later times. Fewer less diverse and reactive T cells infiltrated the metastases nearest to the primary tumor, and a set of prevalent T cell clonotypes were excluded from left-side thoracic tumors further suggesting immune escape near the primary site. Furthermore, shared predicted neoantigens were associated with homology of the T cell repertoire across metastases. Lastly, ratio of methylated neoantigen coding mutations was negatively associated with T-cell density, richness and clonality, suggesting neoantigen methylation may partially drive immunosuppression. Our study demonstrates heterogeneous genomic and T cell profiles across synchronous metastases and how restriction of unique T cell clonotypes within an individual may differentially shape the genomic and epigenomic landscapes of synchronous lung metastases.

## Supplementary Information

**Additional file 1: Supplementary Figure 1.** Distinct mutational profiles are seen across primary tumor and synchronous metastases. **Supplementary Figure 2.** Distinct T cell repertoire metrics between tumors from patients in localized non-small cell lung cancer (NSCLC, PROSPECT cohort) and our patient. **Supplementary Figure 3.** Distinct T cell repertoire metrics between thoracic and non-thoracic tumors but no differences in relation to lymph node involvement. **Supplementary Figure 4.** Distinct antigenic profiles are associated with left-side thoracic tumors. **Supplementary Figure 5.** A higher TCR Jaccard index (JI) in our patient compared to a previously published multi-region localized non-small cell lung cancer (NSCLC). **Supplementary Figure 6.** Characterization of shared T cell clonotype proportions and percentages by all 20 tumors. **Supplementary Figure 7.** Immune cell fractions in left-side thoracic tumors versus non-left side thoracic tumors. **Supplementary Figure 8.** Positive correlations of CD8 to Treg ratio with TCR metrics. **Supplementary Table S1.** Sample information. **Supplementary Data.** TCR rearrangements.

**Additional file 2.**

## Data Availability

Data available upon request.

## Abbreviations

BiTEs: Bispecific T cell engagers
CNAs: Copy number alterations
CT: Computed tomography
DMRs: Differentially methylated regions
H&E: Hematoxylin and eosin
ITH: Intratumor heterogeneity
JI: Jaccard index
LUAD: Lung adenocarcinoma
MBD: Methyl CpG-binding domain
MRI: Magnetic resonance imaging
SNVs: Single nucleotide variants
SWAN: Subset-quantile within-array normalization
TCR: T cell receptor
WES: Whole-exome sequencing
VAF: Variant allele frequency
